# An age-period-cohort analysis of the difference in smoking prevalence between urban and non-urban areas in Japan (2004–2019)

**DOI:** 10.4178/epih.e2020072

**Published:** 2020-12-01

**Authors:** Tasuku Okui

**Affiliations:** Medical Information Center, Kyushu University Hospital, Fukuoka, Japan

**Keywords:** Cohort effect, Japan, Smoking, Urbanization

## Abstract

**OBJECTIVES:**

This study aimed to conduct an age-period-cohort (APC) analysis of smoking prevalence trends in urban and non-urban areas in Japan.

**METHODS:**

Data on smoking prevalence from 2004 to 2019 were extracted from the Comprehensive Survey of Living Conditions in Japan. Government ordinance-designated cities and special wards in Tokyo were defined as urban areas. The respondents ranged from 20 years to 79 years old, and were grouped in 5-year intervals. Cohorts were defined for each age group of each year, and those born between 1925-1929 and 1995-1999 were examined. We calculated the estimated smoking prevalence for each age, period, and cohort, as well as the smoking prevalence ratio of non-urban areas compared with urban areas from the APC analysis results.

**RESULTS:**

The magnitude of the decrease in the period effect on smoking prevalence was larger in urban areas than in non-urban areas for both men and women. The smoking prevalence ratio for non-urban areas compared with that of urban areas was above 1 for men at most time points, except in the older age groups. In addition, the prevalence ratio between the areas decreased, particularly as age increased. For women, the smoking prevalence ratio in non-urban areas compared to urban areas was below 1 until cohorts born in the 1970s, but the trend reversed thereafter.

**CONCLUSIONS:**

The results of this study suggest that further smoking control and cessation measures are necessary, particularly for older cohorts in urban women and for younger ages in non-urban men.

## INTRODUCTION

Smoking is a major risk factor for multiple diseases, such as ischemic heart disease, stroke, chronic obstructive pulmonary disease, and cancer; overall, it is the largest risk factor for adult mortality from non-communicable diseases in Japan [1,2]. The social burden associated with smoking has increased, and smoking cessation has become a global trend in recent years [3]. In Japan, several smoking cessation measures have been instituted. These include the tobacco tax, which was raised in 2003, 2006, and 2010 [4], and the bans on street smoking enforced in many municipalities [5]. Furthermore, ordinances preventing secondhand smoking have been launched in several prefectures [6], and preventive measures against secondhand smoking have been strengthened.

These measures have resulted in a steady fall in smoking prevalence in Japan, particularly for men. Smoking prevalence among men was over 80% in 1970 [7], and has decreased to approximately 30% in 2016 [8]. For women, a slight decline in smoking prevalence was observed throughout the abovementioned years [7]. However, the rate of the decrease of smoking prevalence shrank in recent years [9], and further smoking cessation measures remain a major public health issue for Japan. There are socioeconomic disparities in smoking prevalence around the world [10,11]. A 2014 national survey found a negative relationship between income and smoking prevalence in Japan [12]. Another survey investigating predictors of smoking cessation found that white-collar employees were more likely to quit smoking than blue-collar workers [13]. However, another report found a higher smoking prevalence among urban women than among those in non-urban areas [14], and studies have found a relationship between urbanization and smoking prevalence in other countries [15]. Urban areas are associated with a higher income or educational background in Japan [16,17], and the relationship between socioeconomic status (SES) and smoking prevalence in Japan is considered to be complex. Although the relationship between urban and non-urban areas depends on age, period, and birth cohort, no analysis of the effects of area of residence has been conducted in Japan. Age-period-cohort (APC) analyses are often conducted to identify age, period, and cohort effects on the incidence of a certain condition or the mortality rate [18]. APC analyses are also used to study the prevalence of various conditions, and have been used to investigate smoking prevalence in other countries [19-22]. APC analyses comparing the differences in smoking prevalence according to SES strata have also been conducted in other countries [22], and the results showed disparities in the decreasing rate of the period effect on smoking prevalence according to SES. By conducting an APC analysis for smoking prevalence by area of residence, we may be able to identify ages or cohorts that are in dire need of smoking cessation measures.

In this study, we conducted an APC analysis to clarify the differences in smoking prevalence for each age, period, and cohort in urban and non-urban areas.

## MATERIALS AND METHODS

### Data

We used data from the Comprehensive Survey of Living Conditions in Japan [8], which sought to ascertain the income and health status of households [23]. This survey investigates the health status of Japanese citizens every 3 years, using over 5,000 districts in Japan chosen by stratified random sampling, and all households in the districts are subject to the survey. The numbers of responses (the response rate) for each of the analyzed years were as follows: 2004, 220,836 households (80%); 2007, 229,821 households (80%); 2010, 228,864 households (79%); 2013, 234,383 households (79%); 2016, 224,208 households (77%); 2019, 217,179 households (72%) [8]. These data have been used in several studies in Japan [24,25]. The subjects received a questionnaire asking about their smoking status, with the question “Do you smoke tobacco?” Respondents could indicate that they smoked every day, that they smoked sometimes, that they had quit smoking (and had not smoked in more than a month), or that they did not smoke. Cigarettes and other types of tobacco products were included. The estimated number of each category in Japan was calculated using the prevalence of each category and the estimated number of household members in the survey [23]. We categorized the data for the estimated number of household members and the estimated number of smokers (smoked every day or sometimes) by age group, gender, and area from 2004 to 2019. The age groups categorized into 5-year intervals from 20-24 years to 75-79 years of age were used. The estimated numbers for each prefecture and designated cities (the special wards in Tokyo and government ordinance-designated municipalities) are publicly available. We used the numbers for the special wards in Tokyo and government ordinance-designated municipalities in 2004 as those of urban areas, as in a previous study [14]. The government ordinance-designated municipalities in 2004 were Sapporo, Sendai, Chiba, Saitama, Yokohama, Kawasaki, Nagoya, Kyoto, Osaka, Kobe, Hiroshima, Kitakyushu, and Fukuoka. Although the data for 2001 are also publicly available, the aggregation method for each age group was different from that of the other years, and the data therefore could not be used for an APC analysis. Those who were 75-79 years old in 2004 (born between 1925 and 1929) were the initial cohort, as they were the oldest birth cohort in the data set. Using a 1-year shift in the birth year, starting from the first cohort, the group aged 20-24 years in 2019 (born between 1995 and 1999) comprised the last cohort. Therefore, a total of 71 birth cohorts (1925-1929, 1926-1930, ... , 1994-1998, 1995-1999) were analyzed.

### Statistical analysis

We calculated smoking prevalence based on age groups in urban and non-urban areas. The estimated number of smokers and household members in non-urban areas was calculated by subtracting the number of households in urban areas from those in all of Japan. Age-standardized smoking prevalence was calculated using the population ratio of the total household members in 2019 as the standard population.

We used a Bayesian binomial APC model for analysis, with *y_ij_* being the estimated number of smokers for the age group *i* (1, ... , I) in year *j* (1, ... , *J*) [26]:

*y_ij_*~Binomial (*n_ij_*, *p_ij_*),

where *n_ij_* denotes the corresponding estimated number of household members, and *p_ij_* denotes the corresponding smoking prevalence. The log odds of the smoking prevalence *η_ij_*= log{*p_ij_*/ (1-*p_ij_*)} become:

*η_ij_*=δ+*α_i_*+*β_j_*+*γ_k_*+*z_ij_*,

where *δ* is the intercept, *α_i_* is the effect of the age group, *β_j_* is the period effect, and *γ_k_* (*k*= 1, ... , *K*) are cohort effects. We added the heterogeneity term *z_ij_* to the equation [26]. *I*, *J*, and *K* are the number of time points for age groups, periods, and cohorts, respectively. To identify each parameter, the sum of each effect was restricted to zero. A first-order random-walk was used as a prior for each effect [26]. The programs for the Bayesian APC model were written by the author using *rstan* software (https://rdrr.io/cran/rstan/). The convergence of the estimated parameters was confirmed based on the values of R-hat. Next, the estimated smoking prevalence was calculated for each age, period, and cohort. For example, the estimated smoking prevalence for age group *i* can be calculated as expit ($\hat{\delta }+{\hat{\alpha }}_{i}$), where $\hat{\delta }$ and $\hat{\alpha }$ are the estimated values for the intercept δ and the age effect *α_i_*, and expit denotes a sigmoid function. Furthermore, the smoking prevalence ratio of non-urban areas and urban areas was calculated for each age group, period, and cohort by dividing the estimated smoking rate of non-urban areas by that of urban areas. To ascertain the fit of the APC model, we calculated the deviance information criterion for the age model, age-period model, and age-cohort model in addition to the APC model.

All statistical analyses were conducted using R version 3.5.1 (https://cran.r-project.org/bin/windows/base/old/3.5.1/).

### Ethics statement

Institutional review board approval was not required because we analyzed data that are available to the public.

## RESULTS

Table 1 shows the smoking prevalence in urban and non-urban areas for each age group and gender from 2004 to 2019. The smoking prevalence tended to decrease as age increased. It dropped sharply from 2004 to 2019 in all age groups in both urban and non-urban men. However, there was a difference in the magnitude of the rate of decrease in the age groups of 35-59 years between the 2 areas. Even though decreases in smoking prevalence were seen in many of the age groups, as well as for women, an increase in smoking prevalence was observed in the age groups of 60-69 years for urban areas, and in the age groups of 55-74 years for nonurban areas. There was also a difference in the magnitude of the decrease in smoking prevalence between the 2 areas in the age groups of 40-59 years.

Table 2 shows the age-standardized smoking prevalence in men and women in urban and non-urban areas from 2004 to 2019. Although the age-standardized smoking prevalence decreased from 2004 to 2019 in both areas for men and women, the magnitude of the decrease was larger for urban areas. Furthermore, although the age-standardized smoking prevalence rate was higher in urban areas in 2004 for women, there were little differences between urban and non-urban areas in 2016 or in 2019. Table 3 shows the results of model fitting. There were little differences in the values of the deviance information criterion among the models, except for the age-period model regarding the data of urban men. To explore the reason for the minimal differences among the models, we scrutinized the estimated values of the heterogeneity term (*z_ij_*) in each model.

**Figure 1.**
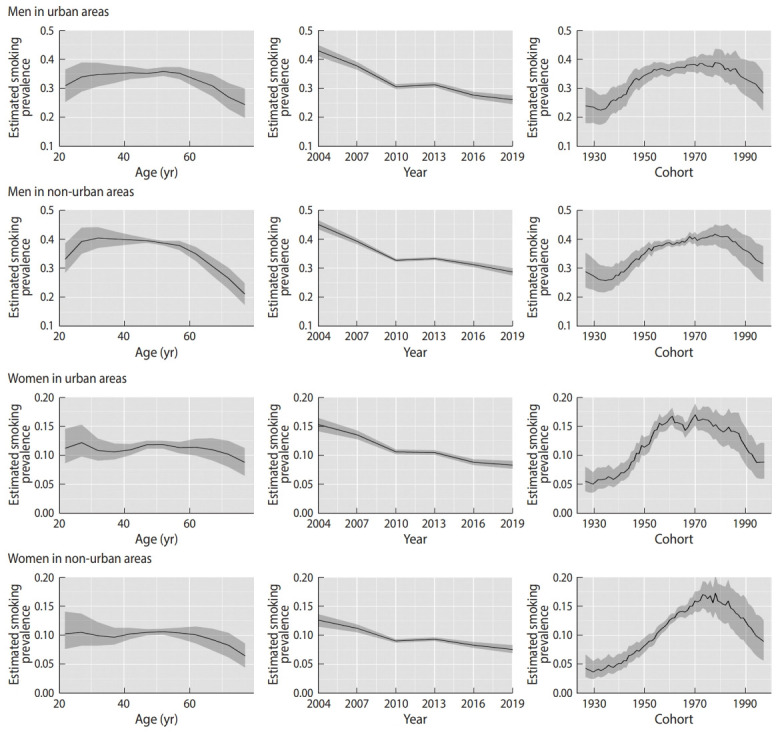
Estimated smoking prevalence for each age, period, and cohort according to gender and area of residence. The graph shows estimated smoking prevalence for each age, period, and cohort in urban and non-urban areas for both genders. Solid lines signify estimates of smoking prevalence, and the shadings show the 95% credible intervals of each effect estimate.

Supplementary Materials 1 and 2 show the estimated values of the heterogeneity term in each model for urban men and women, respectively. On average, the variability of the heterogeneity term was larger in the age and age-period models than in the age-cohort and APC models. In addition, the estimated values of the heterogeneity term for the age model decreased over the periods, and those for the age-period model decreased in young ages and increased in old ages. Therefore, the values of the heterogeneity term in the age and age-cohort models varied by period and birth cohort, and they were not independent and individually distributed. The same phenomenon was also observed for non-urban men and women. Although the model fits for the age-cohort model and APC model were relatively similar, as shown in Figure 1, the estimated smoking prevalence varied depending on the period. Therefore, we present and discuss the results of the APC model.

Figure 1 shows the estimated smoking prevalence for each age group, period, and cohort according to gender and area. The estimated smoking prevalence decreased as the respondents grew older for both urban and non-urban areas in both genders, and particularly, it decreased from the age group of 55-59 years in non-urban men. The estimated smoking prevalence decreased over the years for each gender and area. It increased in cohorts born between the mid-1930s and the mid-1970s, and started falling off thereafter for men. The estimated smoking prevalence for urban women increased from the cohorts born in the middle of 1930s to approximately 1960, but decreased starting in the cohorts born in approximately 1970. In contrast, the estimated smoking prevalence for non-urban women steadily rose from the cohorts born in the 1930s up until the early 1970s.

**Figure 2.**
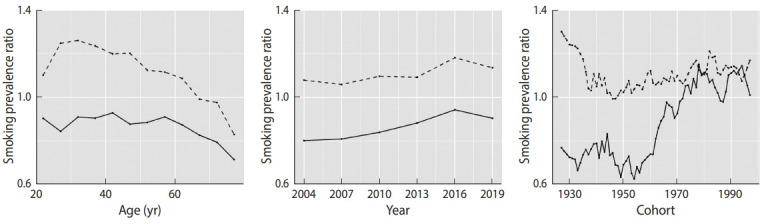
Smoking prevalence ratios of non-urban areas compared with urban areas in each age, period, and cohort for men and women. Solid lines signify estimates of the prevalence ratio for women, and dashed lines signify those for men.

Figure 2 shows the smoking prevalence ratios of non-urban areas and urban areas in each age group, period, and cohort for both genders. The smoking prevalence ratio between the areas was markedly different depending on age, and the smoking prevalence ratio tended to decrease as age increased, particularly for men. In older ages, the ratio was below 1 for men and women. The smoking prevalence ratio also showed an increasing trend from 2004 to 2019 for men and women. The smoking prevalence ratio decreased until cohorts born in the late 1940s for men, and showed a steady increasing trend thereafter. The smoking prevalence ratio for women began to increase in the cohorts born in the 1950s, and showed stationary behavior starting in the cohorts born after the 1970s. Therefore, the relationship between smoking prevalence in urban and non-urban areas was particularly different depending on cohorts for women.

## DISCUSSION

We analyzed differences in smoking prevalence between urban and non-urban areas using data from 2004 to 2019. The results of age-standardized smoking prevalence showed that the difference between urban and non-urban areas decreased in the analyzed period for women. A previous study showed that smoking prevalence was higher in urban areas than in non-urban areas for women in the 18-54 age group in 2001, but not for men [14]. Therefore, the results of the previous study and this study are consistent. Furthermore, this study showed that the relationship between urban and non-urban areas also differed depending on age and cohort.

### Age effect

The relationship between the areas was different depending on age, and the smoking prevalence ratio decreased as age increased in both men and women. In men, the estimated smoking prevalence was higher in non-urban areas for those aged 20-64, before falling in older ages, as shown in Figure 2. These phenomena resulted from differences between urban and non-urban areas in the magnitude of the decrease in smoking prevalence with increasing age. Smoking prevalence tended to decrease with increasing age, as shown in Table 1 and Figure 1, and this phenomenon has been observed in other countries [27-29]. Smoking cessation by older people is one of multiple possible explanations for this phenomenon. A study investigating predictors of smoking cessation in Japan found that age, health check-up participation, and physical activity were predictors [13]. It is also thought that older people quit smoking because they are more conscious of the health risks associated with smoking [29]. Furthermore, a program of specific health check-ups and specific health guidance was started in 2008 in Japan [30], and if a person aged over 40 years has a high-risk of developing a lifestyle-related disease, the person receives specific health guidance on improving their lifestyle behaviors. Therefore, it is possible that the extent of smoking cessation in older ages differed between urban and non-urban areas. As another possibility, there might have been differences in the prevalence of older people who have never smoked between both areas, particularly for women. A survey conducted in 2001 showed that smoking prevalence in urban areas was higher than in non-urban areas for women [14], and it discussed how urbanization and social participation for women tended to increase the likelihood of smoking in Japan [14,31,32]. These factors might account for the difference in smoking prevalence in older women between urban and non-urban areas.

### Period effect

Although the estimated smoking prevalence showed a decreasing trend during the analyzed period for both genders and areas, the magnitude of the decrease was larger in urban areas for both men and women. Although it is known that smoking prevalence is on the decline in Japan [7], differences were found in the rate of decrease according to the region. A difference in the rate of decrease of the smoking prevalence according to SES was also observed in Germany, and some explanations have been proposed [22]. These include low exposure to public health campaigns and a poor ability to process relevant information among people with low SES [22,33]. Smoking-related public health campaigns in Japan were conducted before the analyzed period [34,35], and manufacturers of tobacco products are obliged to attach information on the harmful effects of smoking on their packages [6]. These factors might have led to differences in the perceived risk of smoking between urban and non-urban areas. Another explanation for the observed difference in Germany is that opportunities to smoke may still be greater for low-SES groups, and the impact of smoking bans was different between SES strata [22]. In Japan, tobacco control policies are unevenly implemented across prefectures [36], and there might be differences in smoking cessation measures, particularly between urban and non-urban areas. Furthermore, a lack of social support is also considered to be a causal factor for a lower likelihood of smoking cessation in low-SES groups [10]. Thus, the difference in the number of medical institutions that provide smoking cessation outpatient services between urban and non-urban areas might be another factor contributing to the difference between the areas. Smoking cessation expenses are not high and are covered by medical insurance in Japan [37]. However, the distribution of physicians in Japan is not balanced [38], and access to smoking cessation outpatient services may be relatively limited in non-urban areas.

Regarding the relationship between smoking prevalence and tobacco price increases, it should be noted that tobacco prices and taxes were raised twice during the analyzed period [4], and sales figures dropped significantly in 2010. However, it is not certain to what extent sales changed for low-SES individuals or in non-urban areas, and the results of this study suggest that the effects may have differed depending on the area. Furthermore, data from the Comprehensive Survey of Living Conditions (2007-2010) [39] showed that the increase in tobacco prices had a significant impact on smoking cessation regardless of SES, which is consistent of the results of this study.

### Cohort effect

A significant cohort effect was found for estimated smoking prevalence. An increase in cohort effect for prevalence of smoking was observed starting in the cohorts born in the 1930s in both areas for both genders. In Japan, tobacco consumption per person per day increased from the end of World War II to the 1970s [34], and after 1949, women were targeted in advertising produced by the Japan Tobacco and Salt Public Corporation [7]. Smoking prevalence increased, especially for young women, in this rapid economic growth era, and there was a difference in the time points of an increase in smoking prevalence between regions. The degree of the increase in smoking prevalence was more rapid among non-urban women, and the smoking prevalence ratio increased starting in the cohorts born in the 1960s for women, as shown in Figure 2. Previous studies also have found relationships between urbanization and smoking prevalence for women in other countries [15]. The increase of smoking prevalence across cohorts is considered to have led to a decrease in the smoking prevalence difference between regions for women in Japan. However, estimated smoking prevalence began to fall starting in the cohorts born in the 1970s for men and women. Tobacco consumption in Japan was also shown to decrease from the 1970s [34]. This decline can be attributed to the increase in tobacco prices and public awareness of the dangers of smoking [34]. Several smoking control measures have been introduced since the late 1970s [35]. The smoking prevalence ratio for cohorts born after the 1970s was above 1 for men and women, and smoking prevalence was higher for non-urban areas in younger cohorts.

### Limitation

This study was limited by the absence of data on the smoking prevalence of non-respondents to the Comprehensive Survey of Living Conditions, which had an approximately 80% response rate. It is necessary to conduct a similar analysis using nationwide epidemiological cohort data to check the robustness of the results. Furthermore, although we focused on the difference between urban and non-urban areas (i.e., regional SES), these differences do not necessarily reflect individual or personal SES. By analyzing the difference in smoking prevalence according to individual-level SES, disparities based on SES can be more accurately revealed. Furthermore, we could only obtain the estimated numbers of smokers for each area, and this might have affected the precision of the estimates of the APC analysis. Finally, APC analysis is a descriptive method, and the reasons for the changes in each effect are uncertain. Based on the results of this study, we need to reconsider the reasons for the non-decrease in smoking prevalence in non-urban areas. However, the main strength of this study lies in its use of nationwide national survey data for the analysis, and the results can be generalized to all of Japan.

In conclusion, although the age-standardized smoking prevalence was higher in urban areas than in non-urban areas in 2004 for women, there was almost no difference between the areas in 2019. The APC analysis showed that the smoking prevalence ratio between non-urban and urban areas was significantly affected by age for men. The magnitude of the decrease in the period effects on smoking prevalence was also larger in urban areas than in non-urban areas for men and women. Furthermore, although the smoking prevalence ratio of non-urban areas compared with urban areas was below 1 until the cohorts born in the 1970s for women, the trend reversed thereafter. The smoking prevalence ratio between non-urban and urban areas was significantly affected by age for men and by cohort for women, and smoking cessation measures are needed particularly for older cohorts in urban women and for younger ages in non-urban men.

## Figures and Tables

**Table 1.** Smoking prevalence in urban and non-urban areas for each age group among men and women from 2004 to 2019

Variables	Age, yr											
	20-24	25-29	30-34	35-39	40-44	45-49	50-54	55-59	60-64	65-69	70-74	75-79
Men												
Urban areas												
2004	44.8	46.7	52.1	51.7	51.6	51.0	49.4	48.9	38.2	33.8	25.2	24.7
2007	40.9	46.2	45.1	47.1	46.2	43.5	44.6	42.8	36.6	30.4	21.2	20.7
2010	29.9	37.9	41.1	39.6	39.1	38.3	37.9	35.6	29.7	22.6	20.4	14.5
2013	29.8	38.6	39.8	39.3	37.9	37.3	39.2	38.1	33.9	30.5	20.1	16.4
2016	25.8	29.8	31.2	36.0	35.3	35.6	35.0	33.4	33.0	27.3	21.6	14.7
2019	20.0	27.0	30.5	30.4	34.0	34.9	35.2	31.9	30.8	27.2	21.9	16.1
Non-urban areas												
2004	49.7	55.7	56.1	55.4	53.3	53.0	51.4	46.6	38.4	30.7	26.8	23.7
2007	41.4	50.0	50.9	51.0	49.2	48.3	45.9	42.1	36.0	27.1	21.9	18.6
2010	33.2	43.2	45.4	43.4	44.1	41.4	39.7	36.1	30.4	23.9	17.9	13.7
2013	32.4	42.0	44.7	44.8	42.4	41.7	40.2	39.3	34.2	27.1	20.4	14.1
2016	27.6	36.7	41.5	42.5	41.3	40.4	38.2	37.9	33.9	27.0	19.9	14.1
2019	24.0	32.8	36.7	39.1	39.4	38.1	37.0	34.9	31.2	26.9	20.3	12.9
Women												
Urban areas												
2004	20.4	22.5	20.8	19.0	21.6	22.3	17.5	15.1	10.5	8.8	7.8	6.3
2007	17.7	19.1	19.4	20.7	19.1	21.5	18.5	14.2	11.7	8.0	6.7	4.7
2010	12.3	16.4	14.9	15.4	15.1	16.5	16.0	13.4	9.8	7.1	5.0	4.3
2013	9.9	14.8	13.0	14.6	15.0	14.1	18.0	16.0	11.6	8.4	6.4	4.9
2016	6.8	10.6	11.0	11.4	13.1	14.4	13.6	13.1	12.3	10.0	5.5	3.8
2019	6.7	8.8	10.6	10.3	11.2	13.9	12.5	11.9	12.0	9.0	7.6	3.9
Non-urban areas												
2004	20.9	21.1	20.9	18.4	17.9	16.0	13.0	10.8	8.2	5.9	4.9	3.8
2007	17.7	19.6	19.2	18.7	17.5	16.2	13.4	10.4	8.2	6.0	4.4	2.8
2010	12.8	16.6	17.4	16.4	15.3	14.4	12.0	9.2	7.1	4.9	3.5	2.3
2013	11.9	14.5	15.4	16.9	16.3	14.9	14.0	11.4	8.8	6.5	4.2	3.2
2016	9.0	12.4	13.1	13.9	15.9	14.3	13.7	11.3	8.6	6.5	5.0	2.8
2019	7.3	9.7	11.2	11.9	13.2	14.1	12.7	11.3	9.4	7.0	5.0	2.9

**Table 2.** Age-standardized smoking prevalence in urban and nonurban areas among men and women from 2004 to 2019

Year	Men		Women	
	Urban	Non-urban	Urban	Non-urban
2004	42.5	43.8	15.4	12.5
2007	38.0	39.1	14.6	12.0
2010	31.6	33.5	11.8	10.4
2013	33.0	34.6	12.1	11.1
2016	30.0	33.1	10.6	10.4
2019	28.7	31.1	10.1	9.7

**Table 3.** The result of model fitting

Model	Deviance information criterion			
	Men		Women			
	Urban	Non-urban	Urban	Non-urban
Age	1,149.8	1,232.5	1,101.8	1,172.5
Age-period	1,149.7	NA	1,101.8	1,172.7
Age-cohort	1,150.0	1,233.0	1,101.7	1,172.6
Age-period-cohort	1,149.4	1,232.8	1,101.3	1,172.6

NA, not available.

## Supplementary Materials

Supplementary Material 1. Estimated values of the heterogeneity term in each type of model for urban men

Supplementary Material 2. Estimated values of the heterogeneity term in each type of model for urban women
