# Supplementary material for: An age-period-cohort analysis of the difference in smoking prevalence between urban and non-urban areas in Japan (2004–2019)
Source: Epidemiol Health. 2020 Dec 1;42:e2020072. doi: 10.4178/epih.e2020072 (PMC7871160; doi:10.4178/epih.e2020072)
Supplement: Supplementary Material 2. — Estimated values of the heterogeneity term in each type of model for urban women [file epih-42-e2020072-suppl2.pdf]

Volume: 42, Article ID: e2020072
https://doi.org/10.4178/epih.e2020072

Supplementary Material 2. Estimated values of the heterogeneity term in each type of model for urban women

Periods and age groups	20–24	25–29	30–34	35–39	40–44	45–49	50–54	55–59	60–64	65–69	70–74	75–79
Age model												
2004	0.65	0.56	0.44	0.29	0.40	0.38	0.14	0.12	-0.07	0.03	0.21	0.26
2007	0.47	0.36	0.35	0.40	0.25	0.33	0.21	0.05	0.06	-0.07	0.04	-0.04
2010	0.04	0.17	0.04	0.04	-0.03	0.00	0.03	-0.02	-0.15	-0.20	-0.28	-0.13
2013	-0.20	0.05	-0.13	-0.02	-0.05	-0.18	0.17	0.19	0.04	-0.02	-0.01	0.00
2016	-0.61	-0.33	-0.32	-0.31	-0.20	-0.15	-0.16	-0.04	0.12	0.18	-0.17	-0.27
2019	-0.62	-0.54	-0.35	-0.43	-0.38	-0.19	-0.26	-0.16	0.08	0.06	0.18	-0.23
Age-period model												
2004	0.39	0.28	0.16	0.02	0.13	0.10	-0.14	-0.16	-0.35	-0.23	-0.06	0.02
2007	0.30	0.16	0.17	0.22	0.06	0.13	0.01	-0.14	-0.13	-0.25	-0.14	-0.20
2010	0.07	0.17	0.05	0.06	-0.01	0.00	0.04	-0.02	-0.13	-0.18	-0.26	-0.09
2013	-0.15	0.06	-0.10	0.01	-0.01	-0.16	0.19	0.21	0.07	0.01	0.02	0.06
2016	-0.42	-0.17	-0.15	-0.13	-0.03	0.01	0.01	0.12	0.29	0.36	0.02	-0.06
2019	-0.38	-0.33	-0.13	-0.20	-0.15	0.02	-0.04	0.06	0.31	0.30	0.41	0.03
Age-cohort model												
2004	0.05	0.01	-0.02	-0.01	0.03	0.01	0.00	0.03	0.03	0.02	0.04	0.02
2007	0.03	0.01	0.02	0.05	0.04	0.02	-0.01	0.00	0.04	0.00	-0.01	-0.03
2010	-0.03	-0.01	-0.01	-0.02	-0.03	-0.03	-0.04	-0.02	-0.08	-0.03	-0.07	-0.03
2013	0.00	-0.01	-0.01	0.01	-0.02	-0.04	0.03	0.04	0.00	-0.03	0.02	0.04
2016	-0.04	0.00	-0.02	-0.01	-0.01	-0.01	-0.01	-0.01	0.01	0.04	-0.03	0.00
2019	-0.01	0.02	0.04	-0.02	-0.01	0.04	0.03	-0.03	0.00	0.00	0.05	-0.02
Age-period-cohort model												
2004	0.03	0.00	-0.05	-0.04	0.02	0.00	-0.02	0.01	0.02	0.01	0.03	0.01
2007	0.01	0.00	0.01	0.04	0.03	0.01	-0.03	-0.02	0.04	-0.01	-0.02	-0.05
2010	-0.01	0.04	0.03	0.01	0.00	0.01	0.00	0.01	-0.06	0.00	-0.04	-0.01
2013	-0.02	0.00	-0.02	0.01	-0.03	-0.05	0.05	0.05	0.00	-0.04	0.04	0.04
2016	-0.04	0.00	-0.03	-0.01	0.00	0.01	0.00	-0.01	0.03	0.07	-0.03	-0.01
2019	0.00	0.02	0.03	-0.03	-0.03	0.05	0.03	-0.05	-0.01	-0.01	0.05	-0.05
